# Pulsating fluid flow affects pre‐osteoblast behavior and osteogenic differentiation through production of soluble factors

**DOI:** 10.14814/phy2.14917

**Published:** 2021-06-26

**Authors:** Jianfeng Jin, Hadi Seddiqi, Astrid D. Bakker, Gang Wu, Johanna F. M. Verstappen, Mohammad Haroon, Joannes A. M. Korfage, Behrouz Zandieh‐Doulabi, Arie Werner, Jenneke Klein‐Nulend, Richard T. Jaspers

**Affiliations:** ^1^ Department of Oral Cell Biology Academic Centre for Dentistry Amsterdam (ACTA) University of Amsterdam and Vrije Universiteit Amsterdam Amsterdam Movement Sciences Amsterdam The Netherlands; ^2^ Department of Oral Implantology and Prosthetic Dentistry Academic Centre for Dentistry Amsterdam (ACTA) University of Amsterdam and Vrije Universiteit Amsterdam Amsterdam Movement Sciences Amsterdam The Netherlands; ^3^ Division of Molecular Intensive Care Medicine Department of Anesthesiology and Intensive Care Medicine University Hospital Tuebingen Tübingen Germany; ^4^ Laboratory for Myology Faculty of Behavioral and Movement Sciences Vrije Universiteit Amsterdam Amsterdam Movement Sciences Amsterdam The Netherlands; ^5^ Department of Functional Anatomy Academic Centre for Dentistry Amsterdam (ACTA) University of Amsterdam and Vrije Universiteit Amsterdam Amsterdam Movement Sciences Amsterdam The Netherlands; ^6^ Department of Dental Materials Science Academic Centre for Dentistry Amsterdam (ACTA) University of Amsterdam and Vrije Universiteit Amsterdam Amsterdam The Netherlands

**Keywords:** F‐actin stress fiber, finite element modeling, fluid dynamics, osteogenic differentiation, pre‐osteoblast

## Abstract

Bone mass increases after error‐loading, even in the absence of osteocytes. Loaded osteoblasts may produce a combination of growth factors affecting adjacent osteoblast differentiation. We hypothesized that osteoblasts respond to a single load in the short‐term (minutes) by changing F‐actin stress fiber distribution, in the intermediate‐term (hours) by signaling molecule production, and in the long‐term (days) by differentiation. Furthermore, growth factors produced during and after mechanical loading by pulsating fluid flow (PFF) will affect osteogenic differentiation. MC3T3‐E1 pre‐osteoblasts were either/not stimulated by 60 min PFF (amplitude, 1.0 Pa; frequency, 1 Hz; peak shear stress rate, 6.5 Pa/s) followed by 0–6 h, or 21/28 days of post‐incubation without PFF. Computational analysis revealed that PFF immediately changed distribution and magnitude of fluid dynamics over an adherent pre‐osteoblast inside a parallel‐plate flow chamber (immediate impact). Within 60 min, PFF increased nitric oxide production (5.3‐fold), altered actin distribution, but did not affect cell pseudopodia length and cell orientation (initial downstream impact). PFF transiently stimulated *Fgf2*, *Runx2*, *Ocn*, *Dmp1*, and *Col1⍺1* gene expression between 0 and 6 h after PFF cessation. PFF did not affect alkaline phosphatase nor collagen production after 21 days, but altered mineralization after 28 days. In conclusion, a single bout of PFF with indirect associated release of biochemical factors, stimulates osteoblast differentiation in the long‐term, which may explain enhanced bone formation resulting from mechanical stimuli.

## Graphical abstract

Pulsating fluid flow has distinct temporal impact on pre‐osteoblast behavior and osteogenic differentiation. Initially, PFF increased nitric oxide production, followed in the short‐term by F‐actin stress fiber changes. In the long‐term, PFF did not enhance collagen production, but increased mineralization. This indicates that a single bout of mechanical loading, triggering release of soluble factors, stimulates mineralization in the long‐term.

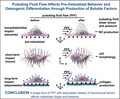

## 1 INTRODUCTION

Bone adapts to mechanical loading. Osteocytes play an important role in this bone adaptation. They are highly mechanosensitive, more so than osteoblasts and fibroblasts. However, osteoblasts also clearly respond, albeit less than osteocytes, to mechanical load by pulsating fluid flow (PFF) and intermittent hydrostatic compression (IHC), for example, with increased prostaglandin E_2_ (PGE_2_) and prostaglandin I_2_ (PGI_2_) release (Klein‐Nulend et al., 1995). PFF also triggers the production of other signaling molecules by osteoblasts that regulate bone mechanical adaptation, such as Wnt signaling (Santos et al., 2009) and nitric oxide (NO) production (Santos et al., 2010). The production of growth factors by osteoblasts may also be altered in response to PFF, such as the production of specific bone morphogenetic proteins and fibroblast growth factors (FGFs), although more research is needed. FGFs play a vital role in the regulation of bone development (Marie, 2003). Fibroblast growth factor‐2 (FGF2) is produced by osteoblasts and stored in the extracellular matrix (ECM) to control osteoblast differentiation (Marie, 2003).

In the absence of loading, the production of sclerostin (osteoblast inhibitor) and RANKL (osteoclast stimulator) by osteocytes increases (Spatz et al., 2015). In this regard, osteocytes can be considered stimulators of unloading‐associated bone loss. Tatsumi et al. showed that hindlimb suspension in osteocyte‐ablated mice did not lead to bone loss, demonstrating the importance of osteocytes for stimulating bone loss with unloading (Tatsumi et al., 2007). However, they also showed that enhanced bone formation after re‐loading did not require osteocytes (Tatsumi et al., 2007). Osteoblasts might regulate loading‐stimulated bone formation independent of osteocytes in the hindlimbs of osteocyte‐less mice (Kwon et al., 2012). This is also in line with reports from the group of Donahue showing that loaded MLO‐Y4 cells hardly affect osteoblast proliferation in co‐culture (Taylor et al., 2007). A limitation is of course that MLO‐Y4 cells do not produce sclerostin, but in the presence of loading primary osteocytes produce little sclerostin anyhow, while it is known that mechanical loading is a potent stimulator of bone (re)modeling by osteoblasts (Papanicolaou et al., 2009). Osteoblasts may either stimulate bone formation by their neighbors through the production of signaling molecules as described above, or the mechanical stimulus directly affects osteogenic differentiation, for example, through alterations in the cytoskeleton.

Mesenchymal stem cells (MSCs) undergo osteogenic differentiation when seeded on a hard substrate, which starts with reorganization of the cytoskeleton and the nuclear skeleton (Engler et al., 2006; Swift et al., 2013). Physical and chemical stimulation affects the organization of the cytoskeleton, resulting in changes in cell adhesion, morphology, and differentiation (McAndrews et al., 2014). Hard substrates affect MSC differentiation, even in the presence of chemical factors (McAndrews et al., 2014). Mechanical loading dramatically changes the orientation of actin stress fibers in MC3T3‐E1 pre‐osteoblasts compared to non‐treated cells (Pavalko et al., 1998). The actin stress fibers become thicker, more abundant, and align roughly parallel to the long axis of the cell (Pavalko et al., 1998). Focal adhesions also can be visualized clearly at the stress fiber termini of the cells treated by mechanical loading (Pavalko et al., 1998). Therefore, our first aim was to map any rapid (seconds, minutes) changes of apex height and fluid dynamics in an osteoblast after a single bout of a precisely defined load.

In this study, our aim was two‐fold: (1) to map any rapid (seconds, minutes) changes of apex height and fluid dynamics over an osteoblast after a single bout of mechanical loading, and (2) to investigate whether osteoblasts produce (a mix of) signaling factors in the intermediate‐term (hours) that affect osteogenic differentiation in the long‐term (days). We showed images of a live cell during mechanical loading (immediate impact), and quantified immediate fluid dynamics over the cell by FE modeling. We tested whether a single bout of mechanical stimulation by PFF modulates the distribution of fluid dynamics over pre‐osteoblasts (immediate impact), signaling molecule (nitric oxide (NO)) production (initial downstream impact), metabolic activity, cell morphology, orientation and expression of osteogenic genes, growth factors, and angiogenesis‐related genes (short‐term downstream impact), as well as alkaline phosphatase (ALP) protein, collagen production and mineralization (long‐term downstream impact). Moreover, biochemical factors released during varying post‐incubation times will have differential long‐term impact on mineralization.

## 2 MATERIALS AND METHODS

### 2.1 Experimental setup

**FIGURE 1:**
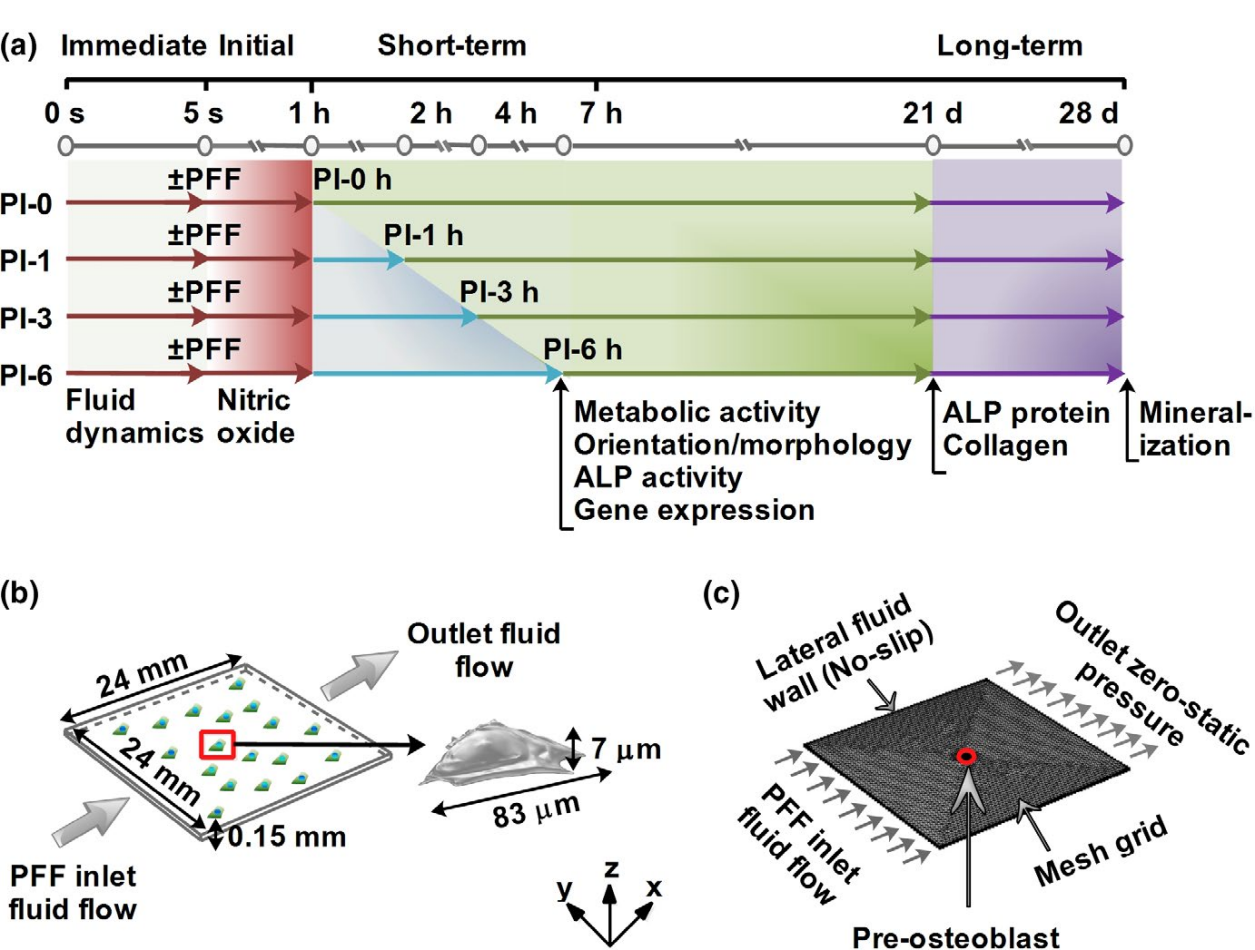
Schematic diagram of the experimental setup. (a) Fluid dynamics (fluid velocity, fluid pressure, and fluid shear stress) over a bone cell as a result of PFF was modeled during 5 s (immediate impact: gray area). NO production as a result of 1 h ± PFF was measured (initial downstream impact: gray and dark red area). Metabolic activity, cell morphology and orientation, and gene expression were assessed after 0, 1, 3, and 6 h post‐incubation (short‐term downstream impact: light blue area). Collagen protein production were assessed after 21 days (long‐term downstream impact: light green area). ECM mineralization was determined after 28 days (long‐term downstream impact: purple area). (b) Schematic illustration of a parallel‐plate flow chamber measuring 24 × 24 × 0.15 mm (length × width × height; total volume, 86.4 mm^3^) used to simulate fluid velocity, fluid pressure, and fluid shear stress, over a bone cell (total volume, 1.2 × 10^−5^ mm^3^; length, 83 μm; height, 7 μm) used in the experimental study. (c) Simulation volume, mesh, and boundary conditions (inlet flow rate, outlet zero static pressure, and no wall slip) applied in the FE modeling. Dark red arrows: 1 h ± PFF; light blue arrows: post‐incubation; green/purple arrows: 21/28 days of culture in osteogenic medium

A schematic diagram of the experimental setup is shown in Figure 1a.

### 2.2 Cell vertical displacement and center of mass

The video of a live bone cell was performed by an SP8 lightning confocal microscope (Leica). Prior to 4 h of PFF treatment, the cells were stained by Sir‐actin (Spirochrome) for F‐actin at 37℃. The flow apparatus with a parallel‐plate flow chamber (1.4 × 1.4 × 0.02 cm^3^) was connected to the confocal microscope. The video of the live bone cell was made for 109 s (before PFF treatment: 0–11 s, PFF treatment: 12–109 s). The video data was analyzed by Image J software (https://imagej.net/Downloads). The quantification of cell vertical displacement was performed as follows: (1) select a rectangle region as the landmark for alignment. (2) Matching method: Normalized correlation coefficient. (3) Search area (pixels around a region of interest (ROI)): 0 (Template will be searched on the whole image if search area = 0). (4) Select subpixel registration (Interpolation method for subpixel translation: Bilinear). (5) Select show align coordinates in results table. Thereafter, the bottom of the cell was selected in the confocal image using the rectangular selection tool for image matching. (6) Measure the center of mass (coM) displacement. “Center of mass” was selected in “Set Measurements” via “Analyse” in Image J software. The whole stack or frames (video) was measured by using “Measure Stack”. In addition, the 60th frame was equal to 11th sec, that is, 1 frame = 0.18 s.

### 2.3 Computational fluid dynamics modeling

#### 2.3.1 Geometry reconstructions

Z‐stack images of a pre‐osteoblast at different time points were imported into a solid modeling software (CATIA V5R21, Dassault Sytemes) to convert the geometries to solid objects (Figure 1b). A three‐dimensional (3D) model of a pre‐osteoblast (total volume, 1.2 × 10^−5^ mm^3^; length, 83 μm; height, 7 μm) and a glass slide (length × width × height, 24 × 24 × 0.15 mm; total volume, 86.4 mm^3^) in the parallel‐plate flow chamber were constructed using commercial finite element software (COMSOL Multiphysics v5.4; Figure 1b) at the same *z*‐coordinates intervals as in the experiments. PFF was initiated by allowing the fluid flow to enter at the left surface, and flow from the left to the right side of the chamber (Figure 1b).

#### 2.3.2 Model assumptions

To model the fluid dynamics on an adherent pre‐osteoblast in a parallel‐plate flow chamber, some assumptions were made about boundary and initial conditions to simplify the FE modeling. The cell was assumed to be attached to the bottom surface of the chamber during the modeling period. The chamber and the attached pre‐osteoblast were assumed to be rigid and not affected by the fluid flow, that is, incompressible and impermeable for fluid. The cell geometry was considered constant during the computational evaluation since PFF did not change the cell topography significantly during the modeling period (5 s). The culture medium inside the chamber was considered as incompressible, and homogeneous Newtonian fluid. The effect of heat dissipation from the culture medium was neglected. Therefore, the culture medium specifications, such as dynamic viscosity and density, were assumed to be constant during the computational analysis.

#### 2.3.3 Laminar fluid flow equations

Time dependent Navier–Stokes equations for incompressible fluid dynamics were used to model a fully developed and laminar flow as described earlier (Saatchi et al., 2019).

#### 2.3.4 Initial and boundary conditions for fluid flow

The initial fluid velocity was set to zero in the simulation volume. The average pressure at the outlet surface of the parallel‐plate flow chamber was set to zero as a boundary condition (Figure 1c). No‐slip boundary condition was applied to the inner surface of the parallel‐plate flow chamber (Figure 1c), while a slip‐boundary condition was applied to the outer surface of the cell (Muha & Čanić, 2016). As inlet flow boundary condition, PFF (inlet flow rate, 3.5 ml/min (small chamber); amplitude, 1.0 Pa; frequency, 1 Hz) was chosen based on the PFF used in the experimental part of our study. The parameters and default values used in the simulation model were as follows: *T* = 37℃ (operational temperature; experimental part of this study), *Q*_inlet_ = 3.5 ml/min × (sin(ω*t) + *b*; *ω* = 2 × *π* × *f*, *f*: frequency, *t*: time, *b*: amplitude; *Q*_inlet_: pulsating inlet flow rate), *ρ* = 893 kg.m^−3^ (culture medium density; Chung et al., 2007), *µ* = 0.83 mPa.s (culture medium dynamic viscosity; Chung et al., 2007), *p *= 1 atm (operation pressure; experimental part of this study).

#### 2.3.5 Mesh generation

For the generation of FE meshes, the “user controlled meshes” with normal element size (total elements: 649554, tetrahedra, 494820; triangles, 150873; edge elements, 3241; vertex elements: 620) was constructed using commercial finite element software. Average element quality was 0.68 measured by skewness, which is considered as good element quality. Maximum mesh element was 0.14 mm, minimum mesh element was 0.00432 mm, maximum element growth rate 1.5, curvature factor 0.6, and resolution of the narrow area 0.5 (Figure 1c).

#### 2.3.6 FE modeling

FE modeling was performed with a time‐dependent fully coupled solver using a commercial FE software package (COMSOL Multiphysics v5.4). An iterative method for the numerical solution of a non‐symmetric system of linear equations (generalized minimal residual algorithm [GMRES]) was used to evaluate the variables fluid velocity, fluid pressure, and fluid shear stress on an adherent pre‐osteoblast inside a parallel‐plate flow chamber.

#### 2.3.7 Average fluid velocity, fluid pressure, and fluid shear stress calculation

The average values of fluid velocity, fluid pressure, and fluid shear stress were evaluated as described previously (Seddiqi et al., 2020).

### 2.4 MC3T3‐E1 pre‐osteoblast culture

MC3T3‐E1 pre‐osteoblasts were cultured in 75 cm^2^ flasks (Nunc) in α–minimal essential medium (α‐MEM, Gibco) supplemented with 10% fetal bovine serum (FBS; Gibco), 300 μg/ml penicillin (Sigma‐Aldrich), 250 μg/ml streptomycin (Sigma‐Aldrich), and 1.25 μg/ml fungizone (Gibco) in a humidified atmosphere of 5% CO_2_ in air at 37℃. The medium was exchanged every 72 h. Upon reaching 80%–90% confluence, cells were harvested using 0.5 mM ethylenediaminetetraacetic acid (EDTA) and 0.25% trypsin (Gibco) for 5 min at 37℃, replated at 1.5 × 10^5^ cells per 75 cm^2^ flask (Greiner Bio‐One), and passaged until the cells reached 80%–90% confluence again. Cells used for PFF experiments were between passage 20 and 29 (P20‐P29).

### 2.5 Pulsating fluid flow

One day before mechanical loading by PFF, MC3T3‐E1 pre‐osteoblasts were seeded at 1 × 10^3^ cells/cm^2^ or 3 × 10^3^ cells/cm^2^ on poly‐L‐lysine‐coated (50 μg/ml; poly‐L‐lysine hydrobromide; Sigma‐Aldrich) glass slides (24 × 24 × 0.15 mm or 36 × 76 × 1 mm). One hour before the start of PFF, the medium was changed by α‐MEM with 2% FBS, 300 μg/ml penicillin, 250 μg/ml streptomycin, and 1.25 μg/ml fungizone. PFF was generated using a flow apparatus containing a parallel‐plate flow chamber (Juffer et al., 2014). A “small chamber” (14 × 14 × 0.2 mm; inner dimension) was used for FE modeling, and measuring metabolic activity, cell orientation, cell morphology, F‐actin fluorescence intensity, ALP activity (short‐term), ALP protein (long‐term), collagen production, and ECM mineralization. A “big chamber” (58 × 32 × 0.3 mm; inner dimension) was used for measuring NO production and gene expression. In both chambers, cells were treated with the same intensity of PFF (amplitude: 1.0 Pa, peak shear stress rate: 6.5 Pa/s, frequency: 1 Hz) for 1 h at 37℃. Static control cultures were kept in a Petri dish under similar conditions as experimental cultures, that is, α‐MEM with 2% FBS, 300 μg/ml penicillin, 250 μg/ml streptomycin, and 1.25 μg/ml fungizone, as well as 1 h incubation at 37℃.

Medium samples of 500 µl were taken at 10, 30, and 60 min of static or PFF treatment, and assayed for NO production. After 60 min of static or PFF treatment, cells were post‐incubated in fresh α‐MEM containing 10% FBS and antibiotics for 1, 3, or 6 h without mechanical loading, and cell morphology, fluorescence intensity, orientation, and metabolic activity were determined, as well as osteogenic, proliferation, and angiogenic gene expression in cell cultures that were lysed in TRIzol^®^ reagent (InVitrogen) for RNA isolation and quantitative real‐time PCR (RT‐PCR; Figure 1a). Some cultures were further incubated in osteogenic induction medium (α‐MEM with 10% FBS, 300 μg/ml penicillin, 250 μg/ml streptomycin, 1.25 μg/ml fungizone, 50 μg/ml ascorbic acid (Sigma), and 10 mM β‐glycerophosphate (Sigma)) for 3 or 4 weeks to determine ALP protein (at 3 weeks), collagen production (at 3 weeks), and ECM mineralization (at 4 weeks; Figure 1a).

### 2.6 Nitric oxide production

Nitric oxide production was measured in medium samples collected at 0, 10, 30, and 60 min of PFF treatment or static culture. Nitric oxide production was measured as nitrite (NO_2_) accumulation in conditioned medium using Griess reagent containing 2.5 mol/L H_3_PO_4_, 0.1% naphtylethelene‐diamine‐dihydrochloride, and 1% sulfanilamide. Serial dilutions of NaNO_2_ in α‐MEM containing 2% FBS were used as a standard curve. The absorbance was monitored at 540 nm with a Synergy HT^®^ spectrophotometer (BioTek Instruments). Four independent experiments with 8 glass slides (*n* = 4) were performed.

### 2.7 MC3T3‐E1 pre‐osteoblast metabolic activity

Cell activity was assessed by using AlamarBlue^®^ Cell Viability Reagent (Invitrogen) at 0, 1, 3, and 6 h of post‐incubation without mechanical loading after 1 h static control or PFF treatment as described above. The cells were incubated with AlamarBlue^®^ reagent in culture medium (1:100) in a humidified incubator with 5% CO_2_ in air at 37℃ for 4 h. After incubation, the supernatants (100 µl/well) were transferred into a 96‐well plate. The absorbance was measured at 450 nm with a Synergy HT^®^ spectrophotometer (BioTek Instruments). Four independent experiments with 28 glass slides (*n* = 4) were performed.

### 2.8 Cell orientation/morphology

To quantify F‐actin fluorescence intensity and cell orientation (angle) using laser scanning confocal microscopy (LSCM; Nikon, A1R/A1), the cells were fixed in 4% paraformaldehyde (Merck) in PHEM buffer containing 60 mM Pipes (Sigma), 25 Mm Hepes (Sigma), 5 mM EGTA (Sigma), 1 mM MgCl_2_ (Merck), 3% sucrose, and 0.1% Triton‐X100 (Serva) for 15 min in the dark at 37℃. After washing for 5 min with PBS, samples were blocked in blocking buffer (PBS containing 5% bovine serum albumin (BSA), 5% glycine, and 0.1% Triton‐X100) for 30 min in the dark. Then, the F‐actinstress fiber was stained using Alexa Fluor 488 (Invitrogen, 1:100) for 40 min at room temperature in the dark. Afterwards, nuclei were stained by using 4’,6‐diamidino‐2‐phenylindole (DAPI; Merck) in PBS (1:1000) for 10 min in the dark at room temperature. Cells were washed gently three times for 15 min with PBS, and mounted in Vecta‐shield (Vector Laboratories) for visualization by LSCM. To measure cell ratio (length/width), Cell images before and after 1 h PFF treatment were taken by normal light microscopy (Leica). Cell F‐actin fluorescence intensity, cell orientation (angle), and cell ratio (length/width) were analyzed using Image‐Pro Plus software (Media Cybernetics). Cell F‐actin fluorescence intensity along the cell long axis was quantified by using the tool “Line profile” in Image‐Pro Plus software. Quantification of cell orientation (angle) and ratio (length/width) were done as follows (1) The tool of “Irregular AOI” was chosen to outline the cell. The parameters of “trace” were set, including thresh = 3, smooth = 0, speed = 5, noise = 5. (2) The tools of “Multiple AOI” “NEW AOI” were used to outline more cells. (3) The parameters of “angle” (between the long axis of the cell and the vertical line), length (major axis of the cell) and width (minor axis of the cell) were selected in the “select measurements” of “Measure”. (4) “Convert AOI(s) To Object(s)” was selected in “Edit” of “Count and measure objects”. (5) “Count” was chosen in the diagram of “Count and size” in “Count and measure objects”. (6) “Measurement data” was chosen in “View” of “Count and measure objects”. (7) The data was saved and used to prepare the figures. To quantify the cell orientation, 169 (control) and 133 (PFF) cells from four glass slides from four independent experiments were analyzed. To quantify the cell ratio, 76 (control) and 80 (PFF) cells from three glass slides from three independent experiments were analyzed.

Scanning electron microscopy (SEM; XL20, Fei Company) was used to visualize static control and PFF‐treated MC3T3‐E1 pre‐osteoblasts at 6 h post‐incubation. Cells were washed with phosphate‐buffered saline (PBS), and fixed with 4% paraformaldehyde (Merck), 1% glutaraldehyde (Merck), and 0.1 M natriumcacodylate at 4℃ overnight. Then the samples were dehydrated in a graded ethanol series (35%, 50%, 70%, 80%, 90%, and 100%), and air‐dried overnight with hexamethyldisilazane (HMDS; Sigma‐Aldrich). To evaluate cell morphology, the specimens were sputter‐coated with gold and examined using SEM at an accelerating voltage of 15 kV. The cells and regions of cells were selected randomly for SEM analysis of pseudopodia. The magnification was ×10,000. Quantification of cell pseudopodia length was performed using Image‐Pro Plus software. The tool of “line” in “Features” of “Manual measurements” was chosen to draw and measure the length of pseudopodia. The data were saved from “Export data” (data to “Features”, output data to “File”) in “Input/Output” of “Manual measurements”. Six cells from 6 glass slides from three independent experiments (*n* = 3; control: 3, PFF: 3) were used for quantification. Five pseudopodia were quantified in every cell.

### 2.9 Alkaline phosphatase activity

ALP activity (short‐term) was measured to assess the osteoblastic phenotype of MC3T3‐E1 pre‐osteoblasts treated with or without 1 h PFF after 0, 1, 3, and 6 h of post‐incubation without mechanical loading (short‐term downstream impact). Cells were lysed with 1.5 ml milli‐Q water, and stored at −20℃ until use. 4‐Nitrophenyl phosphate disodium salt (Merck) at pH 10.3 was used as a substrate for ALP, according to the method as described by Lowry (1955). The absorbance was read at 405 nm with a Synerg HT^®^ spectrophotometer (BioTek Instruments). ALP activity (short‐term) was expressed as μmol/μg cell protein. BCA Protein Assay Reagent Kit (Pierce^TM^) was utilized to measure the amount of protein. The absorbance was read at 540 nm with a Synergy HT^®^ spectrophotometer (BioTek Instruments). Four independent experiments with 32 glass slides (*n* = 4) were performed.

ALP protein (long‐term) was determined after 21 days of culture in osteogenic induction medium (α‐MEM with 10% FBS, 300 μg/ml penicillin, 250 μg/ml streptomycin, 1.25 μg/ml fungizone, 50 μg/ml ascorbic acid, and 10 mM β‐glycerophosphate). The cells were washed three times with PBS, and fixed with 4% paraformaldehyde in PBS for 15 min at 37℃. The BCIP/NBT (5‐bromo‐4‐chloro‐3‐indolyl phosphate (BCIP)/nitro blue tetrazolium (NBT)) phosphatase color development kit (Roche Diagnostics) was used for the colorimetric detection of ALP intensity (long‐term) by incubation for 30 min at 37℃. Quantification of ALP intensity was performed using Image‐Pro Plus software. Note that the images had to be converted to gray scale 8. Three independent experiments providing 72 images from 24 glass slides (*n* = 3) were performed.

### 2.10 Collagen production

Total collagen production by MC3T3‐E1 pre‐osteoblasts attached to the glass slides was visualized and quantified by using picrosirius red stain kit (Chondrex, Inc.). Cells were cultured for 21 days in osteogenic induction medium following 1 h static control or PFF treatment, and post‐incubation (both static control and PFF treatment) in normal culture medium without mechanical loading. Then cells were washed with PBS thrice, and fixed with 4% paraformaldehyde for 15 min at 37℃. Samples were stained for 1 h with picrosirius (0.1 wt%) at room temperature. Then, samples were washed twice with acidified water (5 ml acetic acid/L distilled water) to remove unbound stain, and visualized under a stereo and inverted microscope. For semiquantitative collagen analysis, picrosirius red stain was eluted from the samples using 0.2 M NaOH/methanol (1:1, v/v) for 30 min under shaking. Hundred µl of this solution per well of a 96‐well plate (Greiner Bio‐One) was used to measure the absorbance at 550 nm with a microplate reader (BioRadLaboratories Inc.). A mixed NaOH and methanol solution was used as blank. Three independent experiments with 24 glass slides (*n* = 3) were performed.

### 2.11 ECM mineralization and quantification

Mineralization of the ECM produced by MC3T3‐E1 pre‐osteoblasts attached to glass slides was analyzed after 28 days of culture in osteogenic induction medium, following 1 h PFF or static control treatment. To determine mineralization, cells were washed with PBS, and fixed in 4% paraformaldehyde for 15 min at 37℃. Fixed cells were incubated with 40 mM Alizarin Red staining solution (Merck), pH 4.3, at room temperature for 30 min, and washed extensively with deionized water to remove unreacted dye. Optical images were taken using a stereo microscope. For semiquantitative mineralization analysis, the red‐stained mineralized nodules were dissolved with 10% cetylpyridinium chloride (Sigma) in 10 mM sodium phosphate (Sigma) to measure the optical density at 620 nm. A mixed cetylpyridinium chloride and sodium phosphate solution was used as blank. Three independent experiments with 24 glass slides (*n* = 3) were performed.

### 2.12 Analysis of gene expression

Total RNA was isolated using TRIzol ^®^ reagent (Life Technologies), and stored at −80℃ prior to further use. Complementary DNA (cDNA) synthesis was performed according to the First Strand cDNA Synthesis kit (Thermo Fisher Scientific) in a thermocycler GeneAmp^®^ System 9700 PE (Applied Biosystems). cDNA was stored at −20℃ prior to RT‐PCR analysis, and diluted 5× for gene expression analysis. RT‐PCR reactions were performed using 1 µl cDNA per reaction and LightCycler^®^ 480 SYBR^®^ Green I Mastermix (Roche Diagnostics) in a LightCycler^®^ 480 (Roche Diagnostics). RT‐PCR conditions for all genes were as follows: 10 min pre‐incubation at 95℃, followed by 45 cycles of amplification at 95℃ for 10 s, 56℃ for 5 s, 72℃ for 10 s, and 78℃ for 5 s, after which melting curve analysis was performed. With LightCycler^®^ software (version 1.2), crossing points were assessed and plotted versus the serial dilution of known concentrations of the internal standard. For gene expression analysis, the values of target gene expression were normalized using *Pbgd* (Forward primer sequence (5’‐3’; Forward): AGTGATGAAAGATGGGCAACT; Reverse primer sequence (5’‐3’; Reverse): TCTGGACCATCTTCTTGCTGA) to obtain relative gene expression. RT‐PCR was used to assess expression of the following genes: proliferation marker *Ki‐67* (Forward: CCCTCAGCAAGCCTGAGAA; Reverse: AGAGGCGTATTAGGAGGCAAG), runt‐related transcription factor‐2 (*Runx2*; Forward: ATGCTTCATTCGCCTCAC; Reverse: ACTGCTTGCAGCCTTAAAT), collagen 1⍺1 (*Col1⍺1*; Forward: AACTGGTACATCAGCCCGAA; Reverse: TTCCGTACTCGAACGGGAAT), dentin matrix acidic phosphoglycoprotein‐1 (*Dmp1*; Forward: CGGCTGGTGGACTCTCTAAG; Reverse: CGGGGTCGTCGCTCTGCATC), fibroblast growth factor‐2 (*Fgf2*; Forward: GGCTTCTTCCTGCGCATCCA; Reverse: TCCGTGACCGGTAAGTATTG), matrix extracellular phosphoprotein (*Mepe*; Forward: GGAGCACTCACTACCTGAC; Reverse: TAGGCACTGCCACCATGT), and osteocalcin (*Ocn*; Forward: CAGACACCATGAGGACCATCTT; Reverse: GGTCTGATAGCTCGTCACAA). Three to six independent experiments with 24–40 glass slides (*Ki67* = 40, *Runx2* = 40, *Col1⍺1* = 24, *Dmp1* = 38, *Fgf2* = 40, *Mepe* = 36, *Ocn* = 38; *n* = 3–6; *Ki67* = 3–6, *Runx2* = 3–6, *Col1⍺1* = 3, *Dmp1* = 3–6, *Fgf2* = 3–6, *Mepe* = 3–6, *Ocn* = 3–6) were performed.

### 2.13 Statistical analysis

All data are expressed as mean ± SD from at least three independent, separate experiments. Differences were tested with two‐way analysis of variance, combined with Bonferroni. The independent variables were time (post‐incubation for 0, 1, 3 and 6 h) and treatment (without and with PFF). Differences were considered significant if *p* < 0.05. Statistical analysis was performed using IBM^®^ SPSS^®^ Statistics version 17.0 software package (SPSS Inc.) and GraphPad Prism^®^ 5.0 (GraphPad Software Inc.).

## 3 RESULTS

### 3.1 Immediate impact of PFF (seconds)

#### 3.1.1 Displacement of a live cell treated by PFF

**FIGURE 2:**
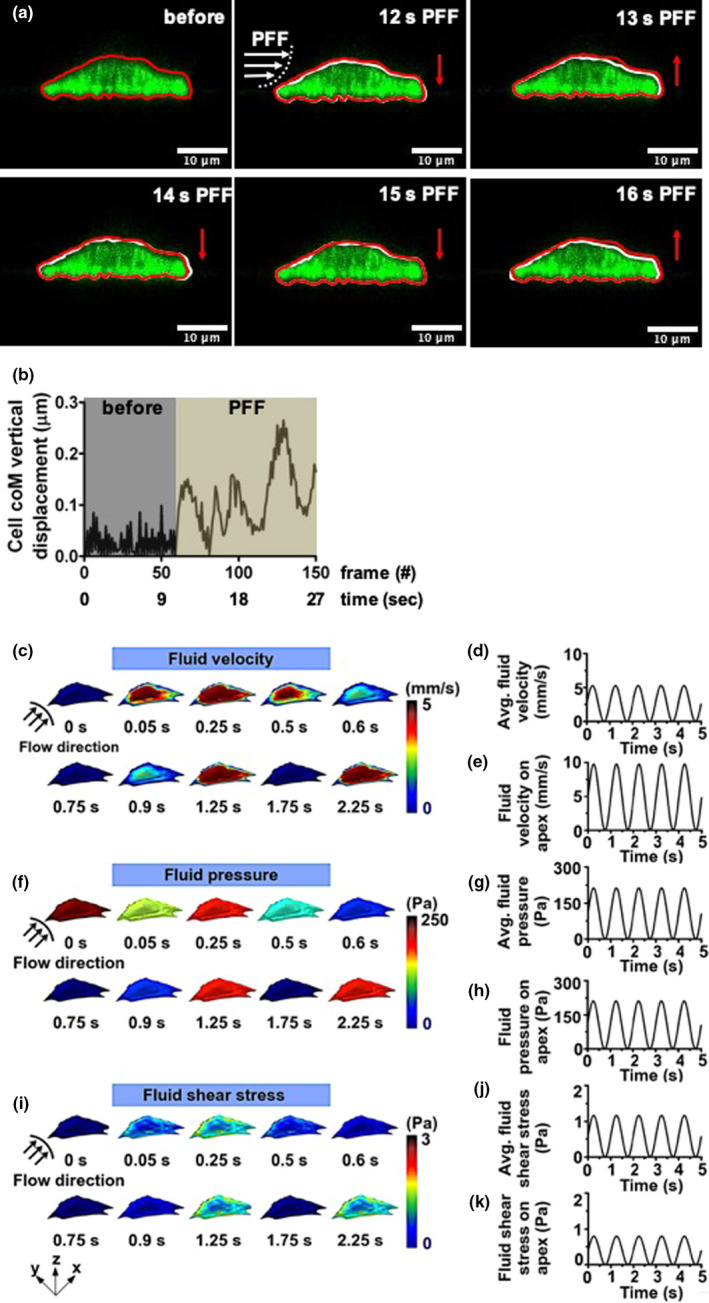
Immediate impact of PFF on cell apex height and distribution of fluid dynamics predicted by FE modeling. (a) The side view of a live bone cell without and with PFF. Red line: cell original shape, white line: cell shape affected by PFF, red arrows: the direction of cell movement during PFF, white arrows: direction of fluid flow, scale bar = 10 μm. (b) Cell coM vertical displacement without and with PFF within 27 s. coM, center of mass. (c) FE modeling of fluid velocity distribution on a pre‐osteoblast illustrated by 3D‐images. The highest fluid velocity magnitude was detected at 0.25 s, and every second thereafter, while the lowest fluid velocity magnitude was observed at 0.75 s, and every second thereafter. (d) The average fluid velocity was oscillating between 0 and 5 mm/s at each pulse. (e) The fluid velocity on the apex was oscillating between 0 and 10 mm/s at each pulse. The magnitude of fluid velocity on the apex of the pre‐osteoblast was 2‐fold higher than the average fluid velocity on the pre‐osteoblast at all time‐points measured. (f) FE modeling of fluid pressure distribution on the pre‐osteoblast illustrated by 3D‐images. The highest fluid pressure magnitude was detected at 0.25 s, and every second thereafter, while the lowest fluid pressure magnitude was observed at 0.75 s, and every second thereafter. (g) The average fluid pressure on the cell was oscillating between 0 and 212 Pa at each pulse. (h) The fluid pressure on the apex of the cell was oscillating between 0 and 212 Pa at each pulse, which was similar to the average fluid pressure on the cell at all time‐points measured. (i) FE modeling of fluid shear stress on the pre‐osteoblast illustrated by 3D‐images. The highest fluid shear stress magnitude was detected at 0.25 s, and every second thereafter, while the lowest fluid shear stress magnitude was observed at 0.75 s, and every second thereafter. (j) The average fluid shear stress on the cell was oscillating between 0 and 1.16 Pa at each pulse. (k) The fluid shear stress on the apex of the cell was oscillating between 0 and 0.8 Pa at each pulse. The magnitude of the average fluid shear stress on the cell was 1.5‐fold higher than the magnitude of fluid shear stress on the apex of the cell all time‐points measured. Black arrows: direction of fluid flow; Surface color: magnitude

Before PFF treatment, the side view of the live cell was oval‐shaped (red line, Figure 2a). During PFF treatment, the cell moved up and down. At the 1st sec PFF (12 s in Figure 2a), the apex height of the cell was decreased (white line, Figure 2a). At the 2nd sec PFF (13 s in the video; Video S1; https://figshare.com/s/10665c52af1d50f443a7; https://doi.org/10.6084/m9.figshare.14386730), the apex height of the cell went back to normal (white line, Figure 2a). At the 3rd and 4th sec PFF (13 and 14 s in Figure 2a), the apex height of the cell was decreased again (white line, Figure 2a). At the 5th sec PFF (16 s in Figure 2a), the apex height of the cell was increased to normal height (white line, Figure 2a). Cell CoM vertical displacement (*y*‐axis) of the PFF‐treated cell occurred within 27 s (Figure 2b). Before PFF treatment, the displacement of the cell ranged from 0 to 0.1 μm (0–11 s, Figure 2b). PFF treatment changed the range of displacement of the live cell from 0 to 0.3 μm (12–27 s, Figure 2b).

### 3.2 Finite element modeling

We performed finite element (FE) modeling of fluid dynamics inside a parallel‐plate flow chamber containing an adherent pre‐osteoblast subjected to PFF during 5 s to assess the dynamics of fluid velocity, fluid pressure, and fluid shear stress over time on the cell. The fluid velocity distribution on the pre‐osteoblast cell membrane due to PFF was non‐uniform and changed over time (Figure 2c). The highest average fluid velocity magnitude (5.27 mm/s) was detected at 0.25 s and every second thereafter, while the lowest average fluid velocity magnitude (0 mm/s) was observed at 0.75 s and every second thereafter (Figure 2d). The average fluid velocity was oscillating between 0 and 5.27 mm/s at each pulse (1 s). At the apex of the pre‐osteoblast, the fluid velocity was oscillating between 0 and 9.7 mm/s at each pulse (Figure 2e). The magnitude of fluid velocity on the apex of the pre‐osteoblast was ∼ 2‐fold higher than the average fluid velocity on the pre‐osteoblast at all time‐points measured (Figure 2e).

The fluid pressure distribution on a pre‐osteoblast cell membrane due to PFF was uniform and changed over time (Figure 2f). Moreover, the fluctuation in fluid pressure on the pre‐osteoblast over time showed that the fluid pressure was highest (212 Pa) at 0.25 s and every second thereafter, while the lowest fluid pressure magnitude (0 Pa) was observed at 0.75 s and every second thereafter (Figure 2g). The average fluid pressure on the cell was oscillating between 0 and 212 Pa at each pulse (1 s; Figure 2g). The fluid pressure on the apex of the cell was oscillating between 0 and 212 Pa at each pulse, which was similar to the average fluid pressure on the cell at all time‐points measured (Figure 2h).

The distribution of fluid shear stress over the pre‐osteoblast was non‐uniform (Figure 2i). The average fluid shear stress over time reached the highest value (1.17 Pa) at 0.25 s and every second thereafter, while the lowest average fluid shear stress magnitude (0 Pa) was observed at 0.75 s and every second thereafter (Figure 2j). The average fluid shear stress on the cell was oscillating between 0 and 1.17 Pa at each pulse (Figure 2j). The fluid shear stress on the apex of the cell was oscillating between 0 and 0.79 Pa at each pulse (Figure 2k). The magnitude of the average fluid shear stress on the cell was ∼ 1.5‐fold higher than the magnitude of fluid shear stress on the apex of the cell all time points measured. These results show that the fluid velocity, and fluid shear stress magnitude varied at different regions of the pre‐osteoblast over time as a result of PFF, while fluid pressure magnitude was constant at different regions of the pre‐osteoblast and changed over time as a result of PFF.

### 3.3 Initial downstream impact of PFF (minutes)

#### 3.3.1 NO production

**FIGURE 3:**
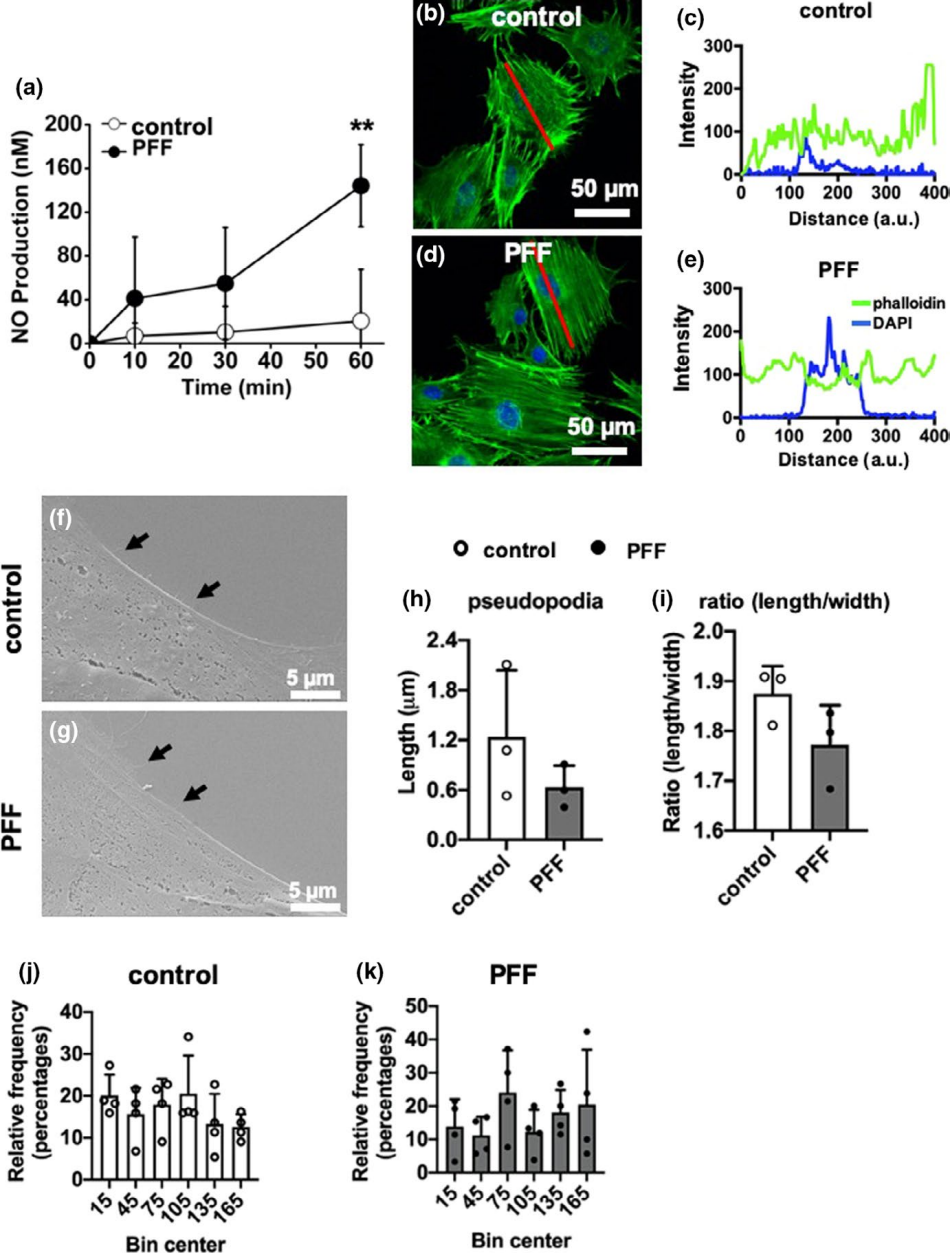
Initial downstream impact of PFF on MC3T3‐E1 pre‐osteoblast behavior. (a) Initial downstream impact of PFF on NO production by MC3T3‐E1 pre‐osteoblasts. Values are mean ± SD. **Significant effect of PFF, *p* < 0.001. *n* = 6. (b, d) Confocal images showing the morphology (F‐actin) of MC3TC‐E1 pre‐osteoblasts without and with PFF. Green: F‐actin, Blue: nuclei. (c, e) F‐actin fluorescence intensity profiles over representative static control cell and PFF‐treated cell, measured along cell long axis (red line) in (b) and (d), respectively. Scale bar = 50 μm. (f, g) SEM pictures showing the pseudopodia formation by MC3T3‐E1 pre‐osteoblast without and with PFF. Black arrows indicate the pseudopodia. Scale bare = 5 μm. (h) Quantification of pseudopodia length in static control cells and PFF‐treated cells. (i) Quantification of cell ratio (length/width) in static control cells and PFF‐treated cells. (j) Histogram of frequency distribution of cell orientation of static control cells. (f) Histogram of frequency distribution of cell orientation of PFF‐treated cells

The absolute amount of NO production by MC3T3‐E1 pre‐osteoblasts in response to PFF was significantly increased after 60 min (5.3‐fold), but not after 10 min (6.0‐fold) and 30 min (4.0‐fold; Figure 3a). Two‐way ANOVA revealed a significant interaction effect between the different post‐incubation times (0, 1, 3, and 6 h; *p* = 0.0014).

### 3.4 F‐actin fluorescence

MC3T3‐E1 pre‐osteoblasts without and with PFF were spread well on glass slides. The morphology of the cells, without and with PFF, was oval‐shaped to more polygonal‐shaped (Figure 3b,d). F‐actin fluorescence (green, static control) intensity was 88.14 ± 46.22 arbitrary units (a.u.; mean ± SD; Figure 3c). PFF‐stimulated F‐actin fluorescence intensity was 105.16 ± 34.21 a.u. (mean ± SD; Figure 3e).

### 3.5 Cell morphology

Control and PFF‐treated cells attached to the glass slides showed protruding filamentous pseudopodia and were spread well (Figure 3f,g). Cells displayed very few pseudopodia in static control and 1 h PFF treatment (black arrows). The length of the pseudopodia was not significantly affected by 1 h PFF (Figure 3h). PFF modulated the shape of the cell body, that is, cell bodies (PFF) seemed more elliptical (ratio of length/width = 1.85 ± 0.13) compared to the static control cells (ratio of length/width = 1.74 ± 0.20; Figure 3i).

### 3.6 Cell orientation

Bin center and percentile were employed to describe potential similarity or differentia of cell orientation between static control and PFF‐treated cells (Figure 3j,k). Each angle (between the long axis of the cell and the vertical line) was approximately equally likely to occur, both in the static and PFF‐treated cells. The direction of the length axis of the cells was randomly distributed in static cells as well as in cells exposed to PFF.

### 3.7 Short‐term downstream impact of PFF (hours)

#### 3.7.1 Cell metabolic activity and ALP activity

**FIGURE 4:**
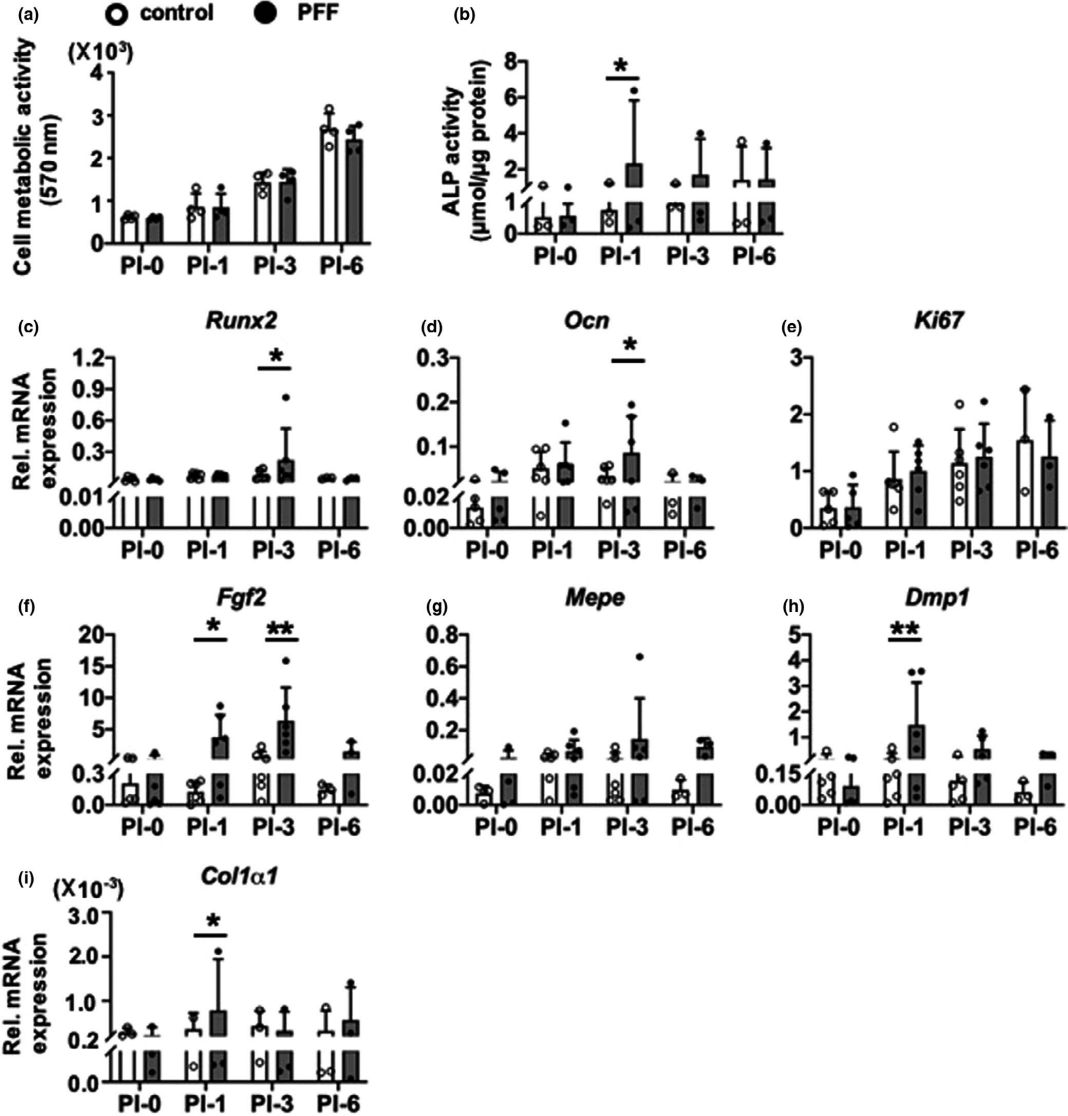
Short‐term downstream impact of PFF on MC3T3‐E1 pre‐osteoblast metabolic activity and gene expression. (a) Short‐term downstream impact of PFF on MC3T3‐E1 pre‐osteoblast metabolic activity at 0, 1, 3, and 6 h of post‐incubation without mechanical loading. (b–h) Short‐term downstream impact of PFF on expression of osteogenic (*Runx2*, *Ocn*, *Mepe*, *Dmp1*, *Col1α1*), proliferation (*Ki67*), and angiogenic‐related (*Fgf2*) genes. (b) *Runx2*, (c) *Ocn*, (d) *Ki67*, (e) *Fgf2*, (f) *Mepe*, (g) *Dmp1*, and (h) *Col1α1* gene expression by MC3T3‐E1 pre‐osteoblasts treated with or without 1 h PFF at 0, 1, 3, and 6 h of post‐incubation without mechanical loading. Values are normalized to *Pbgd* expression. Values are mean ± SD. *Significant effect of PFF, *p* < 0.05, **p < 0.01. *n* = 3–6

Control cell metabolic activity did not change from 1 to 6 h post‐incubation (Figure 4a). One hour PFF‐treatment followed by different post‐incubation periods also did not alter cell metabolic activity compared to untreated controls at all time‐points measured, that is, at 0, 1, 3, and 6 h of post‐incubation without mechanical loading (Figure 4a). Two‐way ANOVA analysis showed that there was a significant interaction effect between without and with PFF treatment (Table 1).

**TABLE 1:** Two‐way analysis of variance for short‐term downstream impact of PFF

Two‐way analysis of variance	Cell activity	ALP activity	*Runx2*	*Ocn*	*Ki67*	*Fgf2*	*Dmp1*
Source of variation	*p* value	*p* value	*p* value	*p* value	*p* value	*p* value	*p* value
Interaction	.8956	.1308	.0663	.1634	.6514	.0241	.0076
Row factor	.0001	.3583	.1343	.0245	.0001	.0088	.0201
Time	.8162	.0306	.0319	.0553	.9601	.0005	.0046

Note

Independent variables: time (post‐incubation for 0, 1, 3 and 6 h) and treatment (without and with PFF).

Treatment with 1 h PFF elicited a significant increase in ALP activity at 1 h post‐incubation, but not thereafter (Figure 4b). Two‐way ANOVA showed that there was a significant interaction effect between post‐incubation time (0, 1, 3, and 6 h; Table 1).

### 3.8 Gene expression

The expression levels of proliferation marker gene *Ki67*, and osteogenesis‐related genes including *Runx2*, *Ocn*, *Fgf2*, *Mepe*, *Dmp1*, and *Col1α1* were assessed in static control and 1 h PFF‐treated MC3T3‐E1 pre‐osteoblasts (Figure 4). PFF increased gene expression of osteogenic markers *Runx2* and *Ocn* at 3 h post‐incubation (Figure 4c,d). However, PFF did not change gene expression of the proliferation marker *Ki67* after 1 h PFF and at 1, 3, and 6 h post‐incubation (Figure 4e). PFF significantly increased *Fgf2* mRNA levels at 1 and 3 h post‐incubation (Figure 4f), but did not affect Mepe mRNA levels at 0, 1, 3, and 6 h post‐incubation (Figure 4g). One h PFF increased *Dmp1* (Figure 4h) and *Col1⍺1* mRNA levels after 1 h post‐incubation (Figure 4i). Two‐way ANOVA revealed significant interaction effects between without and with PFF treatment (*Runx2*, *Ocn*, *Ki67*, *Fgf2*, and *Dmp1*), and post‐incubation time (0, 1, 3, and 6 h; *Fgf2* and *Dmp1*; Table 1).

### 3.9 Long‐term downstream impact of PFF (days)

#### 3.9.1 ALP protein (long‐term)

**FIGURE 5:**
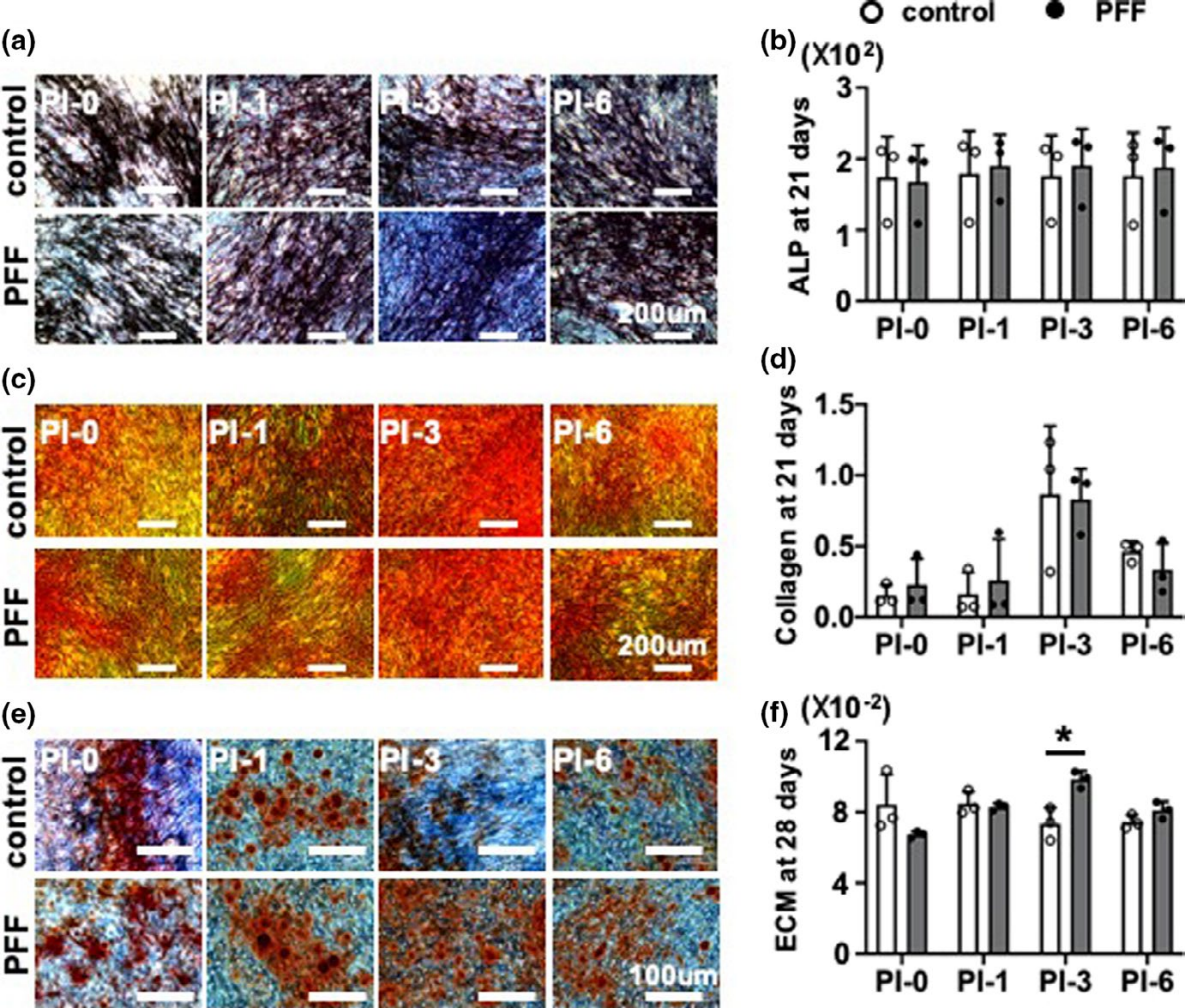
Long‐term downstream impact of PFF on osteogenic differentiation of MC3T3‐E1 pre‐osteoblasts. ALP protein and collagen production were assessed after 21 days of culture, and ECM mineralization after 28 days of culture in osteogenic differentiation medium. After 1 h PFF, the medium was refreshed after 0, 1, 3, 6 h post‐incubation without loading. (a) ALP staining of pre‐osteoblasts. Scale bar = 200 μm. (b) Quantitative colorimetric results of ALP staining. (c) Collagen staining of pre‐osteoblasts. Scale bar = 200 μm. (d) Quantitative colorimetric results of collagen production. (e) ECM mineralization. Scale bar = 100 μm. (ft) Quantitative colorimetric results of ECM mineralization. Values are mean ± SD. *Significant effect of PFF, *p* < 0.05, *n* = 3

ALP protein was quantified in control and 1 h PFF‐treated MC3T3‐E1 pre‐osteoblast cultures after 21 days (Figure 5a,b). PFF did not affect ALP protein in cells that were post‐incubated without medium refreshment for 1, 3, or 6 h compared to static controls and similar post‐incubation conditions (Figure 5a,b).

### 3.10 Collagen secretion

Collagen secretion by MC3T3‐E1 pre‐osteoblasts treated without or with PFF was visualized by picrosirius red staining using light microscopy, and quantified at day 21 (Figure 5c,d). The images showed abundant collagen secretion by cells that were post‐incubated without medium refreshment for 0, 1, 3, and 6 h, after 21 days of culture as shown by the intense red color. PFF did not stimulate collagen secretion compared to static control (Figure 5c,d).

### 3.11 Matrix mineralization

Images of the alizarin red‐stained cultures showed cellular calcium deposition resulting in ECM mineralization (Figure 5e). Static control cultures showed slightly more red‐stained mineralization dots (no significance) than PFF‐treated cultures at 0 h post‐incubation. Interestingly, PFF increased matrix mineralization in cells that were post‐incubated for 3 h, as shown by the presence of abundant mineralization nodules compared to static control cultures (Figure 5e). Quantification of the ECM mineralization levels confirmed this observation, that is, PFF significantly increased mineral deposition in cells post‐incubated for 3 h (Figure 5f).

## 4 DISCUSSION

This study aimed to map any rapid (seconds, minutes) changes of apex height and fluid dynamics over an osteoblast after a single bout of mechanical loading, and investigate whether osteoblasts produce (a mix of) signaling factors in the intermediate‐term (hours) that affect osteogenic differentiation in the long‐term (days). Computational FE analysis revealed that fluid dynamics (fluid velocity, fluid pressure, and fluid shear stress) on an adherent pre‐osteoblast inside a parallel‐plate flow chamber immediately changed as a result of PFF. This data was in line with the movement of the cell in vertical direction during loading by PFF. We also found that PFF considerably affected NO production, slightly affected actin stress fibers, but did not affect cell orientation, or the length of pseudopodia after 1 h PFF. In the short‐term, PFF did not alter cell metabolic activity, but enhanced ALP activity and osteogenic gene expression, that is, it caused a significant increase in *Runx2*, *Ocn*, *Fgf2*, *Dmp1*, and *Col1⍺1* expression. In the long‐term, PFF did not affect ALP and collagen protein production, but changed matrix mineralization, dependent on the post‐incubation time that the cells had experienced without mechanical loading and refreshment of medium. These results indicate that a single bout of mechanical loading by PFF acutely affected signaling molecule gene expression. The observation that PFF treatment followed by post‐incubation in culture medium up to 3 h enhanced the differentiation of the cells in the long‐term (i.e., at 28 days) indicates that the PFF‐induced release of soluble factors had long lasting effects on osteoblast differentiation.

We treated the cells with PFF of 6.5 Pa/s peak shear stress rate, 1.0 Pa amplitude, and 1 Hz frequency. PFF of 6.5 Pa/s peak shear stress rate was used to treat the cells, since we have found earlier that the response of MC3T3‐E1 pre‐osteoblasts is linearly dependent on the rate of fluid shear stress, which depends on the amplitude and frequency of stress (Bacabac et al., 2004, 2005). The fluid shear stress amplitudes and frequencies in bone have been determined by a combination of experiments and computer models, where the peak fluid shear stress around mouse osteocytes in situ has been estimated to range up to 5 Pa (Gardinier et al., 2009). That this range of fluid shear stress is enough to stimulate bone cells was confirmed by in vitro studies (Fahlgren et al., 2018; Klein‐Nulend et al., 1995). In the current study, we have tested the effect of shear stress of a single magnitude within the physiological range, but not shear stresses resembling disuse or overuse, since this will cause bone cell apoptosis and cell death (Tan et al., 2007). Alterations in osteoblast cytoskeletal structure in response to shear stress occur within minutes (Gardinier et al., 2009; McGarry, Klein‐Nulend, & Prendergast, 2005). Therefore, we have chosen 1 h PFF as an end point for our investigations. Previously, we have shown that post‐incubation might affect the behavior of MLO‐Y4 osteocytes and MC3T3‐E1 pre‐osteoblasts (Juffer et al., 2013). Little is known whether post‐incubation affects pre‐osteoblast function after mechanical loading.

### 4.1 Immediate impact of PFF on pre‐osteoblasts predicted by FE modeling

Direct monitoring of fluid dynamics over cells in a parallel‐plate flow chamber is almost impossible. FE modeling is a useful tool to obtain insight in local fluid dynamics for mechanobiological systems (Cao et al., 2011; Zahedmanesh & Lally, 2012). The modeling produces detailed quantitative spatial and temporal information of fluid velocity, fluid shear stress, and fluid pressure exerted directly by fluid flow acting on cells under dynamic conditions (Lesman et al., 2010; Sandino et al., 2008). The level of insight offered by the modeling cannot be obtained by means of experiments alone.

Earlier we found that static fluid flow immediately, within seconds, causes pre‐osteoblast deformation (Jin et al., 2019). Furthermore, static fluid flow has been shown to decrease the apex height of myoblasts, that is, the apex height decreases initially and returns to its original height (Boers et al., 2018). This data is consistent with our finding showing osteoblast movement in vertical direction. To obtain information on the immediate impact of PFF on pre‐osteoblast deformation, FE modeling was employed to analyze the distribution and magnitude of fluid velocity, fluid pressure, and fluid shear stress on the cell membrane. FE modeling confirmed that PFF immediately affected the distribution and magnitude of fluid velocity, fluid pressure, and fluid shear stress over an adherent pre‐osteoblast during 5 s. Our results agree with previous studies suggesting that PFF has an immediate effect on fluid dynamics inside a parallel‐plate flow chamber (Bacabac et al., 2005; Nauman et al., 1999). We found, similar to others (Nauman et al., 1999; Van Kooten et al., 1993), that fluid dynamics over a pre‐osteoblast was oscillating between 0 and 5 mm/s at each pulse. The decrease in fluid pressure on an adherent pre‐osteoblast after a few seconds of PFF can be explained by Bernoulli's principle suggesting that along a horizontal fluid flow, points of high fluid speed have low fluid pressure and vice versa. Therefore, the highest fluid pressure on the pre‐osteoblast was observed at time‐point zero, when PFF did not yet enter the chamber, while the fluid pressure on the cell decreased after PFF started. The fluid velocity and fluid shear stress were also induced on the pre‐osteoblast in the first time fraction after starting PFF, and then oscillated at each pulse. We found that fluid velocity, fluid pressure, and fluid shear stress distribution and magnitude over an adherent pre‐osteoblast varied over time as a result of PFF. A model presented by Chen et al. showed that force application along a different cell axis results in a different cellular volume regulation response (Chen et al., 2019). In our study, we showed that PFF altered the displacement of the live cell from 0–0.1 μm to 0–0.3 μm. In addition, based on the experimental cell deformation results (live cell video; Supplemental Video S1; https://figshare.com/s/10665c52af1d50f443a7; https://doi.org/10.6084/m9.figshare.14386730), we assumed that the glycocalyx of a pre‐osteoblast is very flexible, since the cell deformed within a second after the start of PFF. We implicitly assumed that there were no viscous effects at the cell membrane and thus no boundary layer developed. Thus, the slip boundary condition was applied over the cell in the parallel‐plate flow chamber. Flow controls cell behavior through numerous signaling pathways. Specific links between fluid flow, gene expression, and morphogenesis need to be better understood, but we are just starting to uncover the complexity of interactions between fluid flow and cells. More research is needed to fully understand the immediate physiological response of pre‐osteoblasts to physical loads as a result of PFF resulting in long‐term bone adaptation to mechanical loading.

### 4.2 Initial downstream impact of PFF on NO production and cell morphology

In this study, 1 h PFF stimulated NO production. NO has biphasic effects on bone cells in vitro. High NO concentrations (>30 μM) inhibit cell proliferation, differentiation, and survival, whereas low NO concentrations derived from sodium nitroprusside (SNP, 1 μM) have opposite effects (Holliday et al., 1997; Kalyanaraman et al., 2017; Ralston et al., 2009). Low concentrations of NO derived from SNP (100 μM) induce early mineralization via activation of ALP in rat bone marrow MSCs (Abnosi & Pari, 2019). In addition, mechanical loading‐upregulated NO production stimulates osteogenic differentiation of MSCs after 7, 14, and 21 days, for example, increased ALP activity, collagen synthesis, and mineralization in vitro (Ocarino et al., 2008). NO also significantly contributes to the activation of *Fgf2* expression during angiogenesis (Ziche & Morbidelli, 2000), which is prerequisite for new bone formation. In our study, the initial downstream impact of PFF‐upregulated NO production from 10 to 150 nM, which concentration, albeit low, is still higher than the NO produced by the static control cells. PFF‐upregulated NO production might have stimulated *Fgf2* expression in the short‐term, which could have contributed to the increased ECM production in the long‐term. On the other hand, during post‐incubation, NO induced by PFF could lead to production of another critical soluble factor. Our previous work showed that NO production was increased in a single MC3T3‐E1 pre‐osteoblast from 0 to 90 min post‐incubation after 1 min mechanical stimulation (Vatsa et al., 2006). Future study should measure NO soluble factor in the culture medium after post‐incubation.

Mechanical loading stimulates expression of cyclooxygenase 2 (COX2), a key enzyme for PGE_2_ production in bone cells (McGarry, Klein‐Nulend, Mullender, et al., 2005). COX2 is involved in the reorganization of the F‐actin stress fibers (McGarry, Klein‐Nulend, Mullender, et al., 2005). Such an F‐actin stress fiber reorganization might provide an explanation of our finding that mechanical loading by PFF affected F‐actin fluorescence intensity in bone cells. Our data are in agreement with findings by others showing that mechanical loading affects the reorganization of actin filaments, for example, prominent F‐actin stress fibers (Pavalko et al., 1998). These cytoskeletal changes might allow nuclear genomic adaptation to fluid shear stress by adjusting cellular morphology in the most force‐efficient shape. On the other hand, in the time frame measured we did not find a shift in cell orientation relative to the direction of the flow.

The attachment of pseudopodia to the ECM is achieved by several types of special adhesions, for example, hemidesmosomes, podosomes, fibrillar adhesions, invadopodia, focal complexes, and focal adhesions (Block et al., 2008). As an active organelle, pseudopodia not only participate in cell migration (Monjo et al., 2008), but also sense instantaneous changes in the environment, which is considered as a sensor of basal material morphology (Cooper et al., 2006). Cells loaded by fluid shear stress experience a larger overturning effect, while strain deriving from the substrate mainly affects cell‐substrate attachment (McGarry, Klein‐Nulend, Mullender, et al., 2005). We found that PFF treatment did not modulate pseudopodia length.

The current study showed that PFF was capable of changing F‐actin fluorescence intensity. This might be explained by F‐actin stress fiber reorganization when the cytomembrane was subjected to fluid shear stress. In addition, we found that although PFF caused a change in the internal structure of the cells, it did not alter the orientation of the cells.

### 4.3 Short‐term downstream impact of PFF on cell metabolic activity, ALP activity, and gene expression

In our study, PFF did not affect cell metabolic activity. However, ALP activity was affected by 1 h post‐incubation after PFF treatment. Mechanical loading enhances ALP activity and ALP mRNA of human bone marrow mesenchymal stem cells (Sittichokechaiwut et al., 2010). However, the loading‐induced difference is 40% higher in ALP activity than that in ALP mRNA (Sittichokechaiwut et al., 2010). Therefore, in our study, we did not measure ALP gene expression. *Runx2* is a crucial transcription factor associated with bone cell differentiation. In the cell cycle exit and entry, *Runx2* has a vital cell proliferation regulatory role in osteoblasts (Lucero et al., 2013). We found that PFF increased *Runx2* mRNA expression at 3 h post‐incubation. This increase might be due to the effects of amino acids, vitamins, lipoic acid, and growth factors in the culture medium taken up by osteoblasts after 1 h PFF treatment at that specific time point (3 h post‐incubation). There is an excellent correlation between *Ki67* expression and cell growth or proliferation (Wiesner et al., 2009). In the present study, 1 h PFF did not influence *Ki67* gene expression, which indicates that PFF may not affect cell proliferation. This result is not agreement with the findings of Wiesner et al (Wiesner et al., 2009). Osteocalcin (*Ocn*) is secreted solely by osteoblasts, and plays an important role in the regulation of bone metabolism (Lee et al., 2007). *Ocn* is involved in calcium ion homeostasis and bone mineralization, similar to *Fgf2*. Fibroblast growth factors (*Fgfs*) affect osteoblast gene expression in a biphasic fashion, depending on the stage of osteoblast maturation (Globus et al., 1989; Pitaru et al., 2009). *Fgf2* can activate osteoblast proliferation and *Ocn* production in immature pre‐osteoblasts (Boudreaux & Towler, 1996). We found no significant difference in *Ocn* expression in static control and PFF‐treated cells without post‐incubation, although we might have expected an upregulation, since mineralization in the long‐term was affected by PFF. Both *Ocn* and *Fgf2* expression were not affected by 1 h PFF treatment without post‐incubation. However, *Ocn* and *Fgf2* expression were affected by PFF after 3 h post‐incubation, which might be the result of factors present in the culture medium at the right time. This indicates that bone cells are indeed able to respond to PFF with expression of key factors that are involved in bone formation, for example, Wnt signaling molecules, which regulate bone adaptation. In our study, we performed additional PCR analysis for *Wnt3a*, *Wnt5a*, *LRP5*, and *LRP6* (unpublished results). *Wnt3a* was not detectable in MC3T3‐E1 pre‐osteoblasts at any time point measured, under any condition. *LRP5* and *LRP6* were quantifiable, but were not affected by PFF at any time point measured. *Wnt5a* was also quantifiable, and increased by 1 h PFF after 1 h post‐incubation, suggesting that bone adaptation regulated by osteoblasts might mainly rely on the Wnt noncanonical pathway, that is, *Wnt5a* (Santos et al., 2009). Axin, a key scaffolding protein, is involved into the Wnt signaling pathway. In the absence of Wnt prontein, β‐catenin interacts directly with Axin (Ji et al., 2019). In the presence of Wnt protein, *LRP5* and *LRP6* co‐receptor bind to the Wnt ligand and stimulate the phosphorylation of *LRP5* and *LRP6* (Ji et al., 2019). When Wnt binds to frizzled as well as LRP5/6 the complex targeting β‐catenin for destruction is recruited to the cell membrane and inactivated, thereby allowing β‐catenin to accumulate and translocate to the nucleus, where it affects the transcription of Wnt target genes by TCF/LEF. One such Wnt target gene is Axin. (Ji et al., 2019; MacDonald et al., 2009). *Mepe* plays a multifunctional role in the regulation of mineral homeostasis, cell signaling, and mineralization (Chrepa et al., 2017). *Mepe* belongs to the small integrin‐binding ligands. In this study, 1 h PFF did not affect *Mepe* gene expression. This suggests that *Mepe* might be not involved in the PFF‐upregulated mineralization in the long‐term. *Dmp1* is a non‐collagenous ECM protein found in dentin and bone, similar as osteopontin and bone sialoprotein, that combines ligand N‐linked glycoprotein family, and is part of the small integrin‐binding ligand (Fisher et al., 2004). Collagen type I secreted by early undifferentiated osteoblast‐like cells or pre‐osteoblasts constitutes 90% of the total organic part of the ECM in mature bone. In this study, 1 h PFF stimulated *Dmp1* and *Col1⍺1* expression after post‐incubation, which was likely as the result of enhanced ALP activity by PFF treatment. Gene upregulation of the osteogenic marker *Dmp1* is generally believed to be positively correlated to the differentiation process. Our finding that PFF upregulated *Dmp1* expression is in line with the upregulation of osteogenic differentiation of MC3T3‐E1 pre‐osteoblasts. Therefore, one single bout of mechanical loading by PFF plays a vital role in the upregulation of *Dmp1* and *Col1⍺1* expression, which is likely responsible for the enhanced cell function or mineralization in the long‐term.

### 4.4 Long‐term downstream impact of PFF and post‐incubation in osteogenic medium

PFF causes stress of all parts of bone cells including increased membrane stress, and thereby a rapid intracellular calcium response, ion channel activity, and/or modulation of NO production (Bakker et al., 2016; McGarry, Klein‐Nulend, Mullender, et al., 2005), which may all affect osteogenic differentiation. During post‐incubation, PFF might have affected nutrient uptake from the culture medium containing non‐essential amino acids, vitamins, biotin, sodium pyruvate, cyanocobalamin, lipoic acid, nucleosides, and growth factors (Stanners et al., 1971), which could have affected cell behavior, for example, osteogenic differentiation. On the other hand, soluble factors produced by the cells themselves as a result of PFF treatment might have affected osteogenic differentiation, for example, via NO and/or *Fgf2*. On the other hand, soluble factors produced by the cells themselves as a result of PFF treatment might have affected osteogenic differentiation, for example, via NO and/or *Fgf2*. A low concentration of NO induces cell proliferation, differentiation, early mineralization, and *Fgf2* expression. *Fgf2* produced by osteoblasts is stored in the ECM to control osteoblast differentiation (Marie, 2003). The results of this study and our previous study (Klein‐Nulend et al., 1995) show that both NO and *Fgf2* were upregulated by PFF, which might affect the process of mineralization. The PFF‐induced release of these soluble factors might have long lasting effects on osteoblast differentiation.

In conclusion, our results indicate that mechanical stimulation in the form of PFF has immediate (within 5 s) impact on fluid dynamics over the cells, and affects pre‐osteoblast morphology in the short‐term, which is associated with enhanced osteogenic differentiation and matrix production by these cells in the long‐term. This indicates that a single bout of mechanical loading with indirect associated release of soluble factors stimulates osteoblast differentiation in the long‐term. Future studies will unravel the underlying mechanism and provide new insights into whether, and which morphological changes as a result of PFF drive osteogenic differentiation.

## CONFLICT OF INTEREST

All authors have stated explicitly that there are not conflicts of interest in connection with this article.

## AUTHOR CONTRIBUTIONS

Concept and design of the research: Jianfeng Jin, Hadi Seddiqi, Astrid D. Bakker, Jenneke Klein‐Nulend and Richard T. Jaspers. Experimental work: Jianfeng Jin, and Hadi Seddiqi. Data analysis, interpretation, and writing: Jianfeng Jin, Hadi Seddiqi, Astrid D. Bakker, Jenneke Klein‐Nulend, and Richard T. Jaspers. Review and editing: Jianfeng Jin, Hadi Seddiqi, Astrid D. Bakker, Gang Wu, Johanna F.M. Verstappen, Mohammad Haroon, Joannes A.M. Korfage, Behrouz Zandieh‐Doulabi, Arie Werner, Jenneke Klein‐Nulend, and Richard T. Jaspers. All authors have approved and agreed with the submitted version.
